# Jenner Monument

**Published:** 1858-01

**Authors:** 


JENNER MONUMENT.—It will be remembered by many of our readers that several years ago subscriptions were made in this country for the purpose of erecting a monument to Jenner, the illustrious discoverer of vaccination. The history of the accumulation of funds wherewith to defray the expenses of this merited tribute is interesting to us as Americans. When it was proposed in England to erect a monument to the memory of this distinguished physician and philanthropist, it was suggested that, as it might be an object of universal interest, the people of all nations would gladly contribute; aid was accordingly solicited from the United States, and the appeal was so much more cordially responded to in this country than in the one in which the movement originated, that it may be truly said that the Jenner Monument in London is essentially an American tribute, which the English people have assisted in paying to an English celebrity. Of the whole sum collected some time ago, *three hundred and forty pounds* were transmitted from America—*one hundred and twelve pounds* from the Philadelphia committee; and in the country of his birth, but *one hundred and ninety-six pounds*, including *twenty-five pounds* from the Prince-Consort.

The following letter exhibits the present financial condition of the undertaking, and gives satisfactory assurances that the work has been properly performed :—

LONDON, 24th November, 1857.

SIR:—The Committee have great pleasure in informing you that the statue of Dr. Jenner, by Mr. Calder Marshall, R.A., has now been completed in bronze, and that it is most satisfactory both as a likeness and a work of art.

They have also much gratification in announcing that her Majesty's Board of Works has graciously granted them a most eligible site in Trafalgar Square, and that they have been enabled to enter into a contract for the furnishing and erecting of the pedestal within four months, at a moderate cost.

The Committee, however, regret to add that their funds are still scarcely sufficient to meet the necessary outlay. They have, in consequence, resolved that the subscription lists shall continue open until the 1st of February next, on which day they will be finally closed.

It is requested that all unpaid subscriptions may be transmitted to Messrs. Coutts & Co., or to the Hon. Secretary, No. 5 St. Mark's Crescent, Regent's Park, London.

I remain your most obedient servant,

GEORGE VERE IRVING,

DR. ROBLEY DUNGLISON. Hon. Secretary.

